# Implementation of two-stage testing for *Clostridioides difficile* at a tertiary referral veterans hospital

**DOI:** 10.1017/ash.2026.10427

**Published:** 2026-06-04

**Authors:** Madeline Marker, Dimitri Drekonja

**Affiliations:** 1 University of Minnesota Medical School, Twin Cities Campus: University of Minnesota, USA; 2 Infectious Disease Section, Minneapolis Veterans Affairs Health Care System, Minneapolis, MN, USA

## Abstract

*Clostridioides difficile* infection is a common community and nosocomial infection. Two-stage testing (initial polymerase chain reaction (PCR) test, followed by a confirmatory toxin test for PCR+ results) has been used to help differentiate colonization from infection. We report the impact of adopting two-stage testing at a tertiary Veterans Affairs medical center.

## Introduction

*Clostridioides difficile* infection (CDI) is a common cause of diarrhea in US hospitals, affecting ∼1% of hospitalized patients each year.^1^ This translates to 453,000 cases/year, 83,000 recurrences, and 29,300 deaths,^2^ with estimated costs of $1 to $4.9 billion annually.^3^

The prevalence and morbidity of CDI make accurate diagnosis and treatment essential. Manifestations range from asymptomatic carriage to mild to severe diarrhea, toxic megacolon, and pseudomembranous colitis.^2^ In the 2000s, highly sensitive polymerase chain reaction (PCR) stool testing for the gene encoding *C. difficile* toxin B became widely adopted as a stand-alone test.^1,4^ However, some patients with positive PCR results (PCR+) had few signs of infection, and when tested for toxin B by enzyme-linked immune assay (ELISA), results were often negative (PCR+/Tox−).^1^ PCR+/Tox− patients had similar outcomes to patients with diarrhea who were PCR−. PCR+/Tox+ patients have worse outcomes.^1^ This observation, and the increase in CDI diagnoses when stand-alone PCR testing was instituted, led to the hypothesis that PCR testing identified colonization with *C. difficile* capable of producing toxin B, but without toxin production. Treatment of carriers confers no known benefit and may be harmful.^5^ Accordingly, two-stage testing has been increasingly used,^6^ whereby all PCR+ samples receive a confirmatory toxin test, with results reported as PCR−, PCR+/Tox−, or PCR+/Tox+.

The Minneapolis Veterans Affairs Health Care System (MVAHCS) implemented two-stage testing for CDI in July 2022 with the expectation that it would decrease unnecessary treatment of PCR+/Tox− patients based on prior studies demonstrating no detectable harm. This study evaluates the impact of two-stage testing on patient management (antibiotic use) and outcomes (symptom resolution, CDI-related complications, ICU care, or death).

## Methods

We performed a retrospective review of adult veterans with PCR+ stool samples for *C. difficile* at the MVAHCS from 7/1/2022 to 9/17/2025. Testing was performed via a platform only for CDI (Cepheid) or testing for multiple bacterial, viral, and protozoal infections (BioFire). Testing was at provider discretion, with guidance in the ordering menu reminding clinicians that testing is appropriate for patients not on laxatives, without another known cause of diarrhea, and at least three diarrheal stools/day. Formed stools were declined per laboratory policy. Only the first positive test per patient was included. All PCR+ stool samples were tested for toxin using an ELISA-based stool antigen test; PCR− samples received no further testing. PCR+ results were reported immediately. Toxin results released ∼60 minutes later. The study protocol was reviewed by the MVAHCS Institutional Review Board (IRB) and categorized as quality improvement exempt from oversight.

Record review was performed by a single person (MM) and discussed with a second team member (DD). Abstracted data included demographic information, age-adjusted Charlson Comorbidity Index (CCI), date of CDI symptom onset, lab values, stool consistency and count, stool testing results, selection and duration of antimicrobials treating CDI, and details of non-CDI antimicrobials. Treatment response and outcomes data included symptom resolution, repeat CDI testing and treatment, CDI-related complications (ie, megacolon and colectomy for fulminant colitis), death within 30 days, and need for ICU-level care.

Statistical analysis was performed based on toxin status. Baseline characteristics, treatment, and outcomes were compared between groups. A *P*-value of <.05 was considered significant.

## Results

Two hundred and forty participants had a PCR+ sample, 83 (34.6%) PCR+/Tox+, and 157 (65.4%) PCR+/Tox− (Table 1). PCR+/Tox+ participants typically had an initial white blood cell count ≥15,000 or <4,000 (40.5% vs 21.8%; *P* = .04) (Table 1). There were no significant between-group differences for other lab values (Table 1). Loose stool on the day of PCR testing was similar between groups (75.9% vs 65.0%; *P* = .11) (Table 1).

PCR+/Tox+ participants were significantly more likely to receive antibiotics (98.8% vs 51.3%; *P* < .001) (Table 2). Fidaxomicin was the most common treatment (61.0% vs 56.4%; *P* = .63). Mean treatment was 10 days for both groups.

Symptom resolution was similar (78.3% vs 77.1%; *P* = .87). PCR+/Tox+ participants had a higher rate of CDI-related complications (4.8% vs 0.6%; *P* = .04) (Table 2). Complication rates did not differ between PCR+/Tox− participants who received antibiotics and those who did not (0.6% vs 0%; *P* = .5) (Table 2). Death or need for ICU care was similar (3.6% vs 6.4%; *P* = .55) and (7.2% vs 8.9%; *P* = .81), respectively (Table 2).

There was no significant difference in participants who received repeat CDI testing within 14 days (1.2% vs 5.7%; *P* = .17). Of those retested, 0 PCR+/Tox+ versus 2 PCR+/Tox− were PCR+/Tox+ on repeat testing (*P* = 1.0). Only 3 of 9 (33.0%) initially PCR+/Tox− underwent treatment, all with fidaxomicin.

PCR+/Tox+ participants were more likely to undergo repeat testing within 15–30 days (15.7% vs 2.6%; *P* = .0003). Of those tested, toxin positive rates were similar (53.9% vs 50.0%; *P* = 1.0). Both participants initially PCR+/Tox− but subsequently PCR+/Tox+ on retesting were treated with vancomycin, compared to 3/7 PCR+/Tox+ (100% vs 42.9%; *P* = .4).

Some participants from both cohorts were treated empirically for recurrent symptoms without CDI testing (6.0% vs 2.5%; *P* = .30).

## Discussion

Most participants with PCR+ results for *C. difficile* lacked detectable toxin (65.4%), highlighting the clinical ambiguity in identifying patients to test. Symptoms are non-specific (abdominal pain, fever, and loose stool). Loose stool was reported at testing in participants with and without detectable toxin (Table 1). Participants with Tox+ results were more likely to have abnormalities in their WBC count, while other laboratory markers for illness severity were similar (Table 1).

Toxin testing significantly influenced CDI treatment. Nearly all PCR+/Tox+ participants received treatment (98.8%) versus 51.2% of PCR+/Tox− participants (Table 2). PCR+/Tox+ participants had a higher rate of CDI-related complications (4.8% vs .6%) (Table 2), suggesting they differ from PCR+/Tox− participants. Despite only 51.2% of PCR+/Tox− participants receiving treatment, complication rates did not differ between those who received antibiotics (.6%) and those who did not (0%). Additionally, the majority in both cohorts reported symptom resolution (78.4% vs 77.1%) (Table 2). This aligns with prior literature finding no increase in adverse events when withholding antibiotics in Tox− patients.^7,8^ The implementation of two-stage testing identified a large proportion of PCR+/Tox− participants, half of whom did not receive treatment, without signal of harm. This suggests two-stage testing can identify a population that may not have CDI and be managed with supportive cares.

These findings have clinical implications. The sensitivity of PCR testing allows detection of patients with small amounts of *C. difficile* genetic material, with studies demonstrating higher CDI diagnosis rates with PCR testing.^1^ Treatment of patients with colonization increases antimicrobial use, disrupts the microbiome, and potentially increases CDI rates.^5^ Although 51% of PCR+/Tox− patients still received treatment, 49% (77 individuals) were spared treatment without discernable harms. Transition to two-stage testing was accompanied by limited education, largely via reminders of appropriate test ordering within the medical record. Limitations include the retrospective and single-center nature of this study, and that participants were largely White, male, and non-Hispanic/Latino.

**Table 1.** Comparison of demographic and initial laboratory data on date of PCR testing between PCR+/Tox+ and PCR+/Tox− cohorts Table 1 long description.

Demographic and laboratory data	PCR+/Tox+ (n = 83)	PCR+/Tox− (n = 157)
Median age (IQR)	74 (67,78)	74 (63,79)
Female sex, No. (%)	5 (6.0)	18 (11.5)
White race, No. (%)	69 (83.1)	127 (80.9)
Not Hispanic or Latino, No. (%)	75 (90.4)	146 (93)
Median CCI (IQR)	5 (4,8)	5 (3,7)
WBC count ≥ 15,000 or <4,000 when symptoms developed, No./No. tested (%)	17/42 (40.5)^*^	19/87 (21.8)^*^
Cr level > 1.5, No./No. tested (%)	6/39 (15.4)	25/89 (28.1)
Albumin, No./No. tested (%)	2/24 (8.3)	7/58 (12.1)
Diarrhea present at testing, No. (%)	63 (75.9)	102 (65.0)
Median stool count at testing (IQR)	3 (2.75,5.25)	3 (2,5)

Note. No., number; IQR, interquartile range.

* *P* ≤ .05.

**Table 2.** Comparison of treatment modalities and patient outcomes between the PCR+/Tox+ and PCR+/Tox− cohorts Table 2 long description.

Treatment modalities and patient outcomes	PCR+/Tox+ (n = 83)	PCR+/Tox− (n = 157)
Treatment with typical antibiotics (oral vancomycin, fidaxomicin, or metronidazole), No. (%)	82 (98.8)^**^	78 (52.3)^**^
Use of non *C. difficile* antibiotic, No. (%)	30 (36.1)	53 (33.8)
Symptom resolution, No. (%)	65 (78.3)	121 (77.1)
Median duration of diarrhea after treatment days (IQR)	7 (4,17)	5 (2,10.5)
CDI-related complications, No. (%)	4 (4.8)^*^	1 (.6)^*^
Death within 30 days of treatment, No. (%)	3 (3.6)	10 (6.4)
ICU-level care, No. (%)	6 (7.2)	14 (8.9)

Note. No., number.

* *P* ≤ .05.

** *P* < .001.

## Data Availability

The data that support the findings of this study are not publicly available due to ethical and privacy restrictions. Specifically, the data include protected health information, and participants did not provide consent for public data sharing.

## Table 1. Long description

The table presents a comparison of demographic and laboratory data between two cohorts: PCR/Tox with eighty-three participants and PCR/Tox with one hundred fifty-seven participants. The data includes median age with interquartile range, number and percentage of females, white race, and non-Hispanic or Latino individuals. It also lists median Charlson Comorbidity Index (CCI) with interquartile range, white blood cell (WBC) count when symptoms developed, creatinine (Cr) level, albumin levels, presence of diarrhea at testing, and median stool count at testing with interquartile range. Notable trends include a higher percentage of females and non-Hispanic or Latino individuals in the PCR/Tox cohort. The PCR/Tox cohort also shows a higher median CCI and a higher percentage of abnormal WBC counts and Cr levels. Diarrhea presence is more common in the PCR/Tox cohort, while median stool counts are similar between the two groups.

Navigate back to Table 1..

## Table 2. Long description

The table compares treatment modalities and patient outcomes between two cohorts: PCR/Tox and PCR/Tox. It consists of seven rows and four columns, including headers. The columns are labeled as Treatment modalities and patient outcomes, PCR/Tox (n=83), and PCR/Tox (n=157). The rows detail various metrics such as treatment with typical antibiotics, use of non-C. difficile antibiotics, symptom resolution, median duration of diarrhea after treatment, CDI-related complications, death within 30 days of treatment, and ICU-level care. Notable trends include a higher percentage of typical antibiotic use in the PCR/Tox cohort (98.8 percent) compared to the PCR/Tox cohort (52.3 percent). The PCR/Tox cohort also shows a lower median duration of diarrhea (5 days) compared to the PCR/Tox cohort (7 days). CDI-related complications are more frequent in the PCR/Tox cohort (4.8 percent) than in the PCR/Tox cohort (0.6 percent). Death within 30 days of treatment is higher in the PCR/Tox cohort (6.4 percent) compared to the PCR/Tox cohort (3.6 percent). ICU-level care is also more common in the PCR/Tox cohort (8.9 percent) than in the PCR/Tox cohort (7.2 percent).

Navigate back to Table 2..

## References

[1] Polage C , Gyorke C , Kennedy M , et al. Overdiagnosis of *Clostridium difficile* infection in the molecular test era. JAMA Intern Med 2015;175:1792–1801. 26348734 10.1001/jamainternmed.2015.4114 PMC4948649

[2] Lessa FC , Mu Y , Bamberg WM , et al. Burden of *Clostridium difficile* infection in the United States. N Engl J Med 2015;372:825–834. 25714160 10.1056/NEJMoa1408913 PMC10966662

[3] Kociolek LK , Gerding DN , Carrico R , et al. Strategies to prevent *Clostridioides difficile* infections in acute-care hospitals: 2022 update. Infect Control Hosp Epidemiol 2023;44:527–549. 37042243 10.1017/ice.2023.18 PMC10917144

[4] Prosty C , Hanula R , Katergi K , Longtin Y , McDonald EG , Lee TC. Clinical outcomes and management of NAAT-positive/toxin-negative *Clostridioides difficile* infection: a systematic review and meta-analysis. Clin Infect Dis 2024;78:430–438. 37648251 10.1093/cid/ciad523

[5] Johnson, S , Homann, SR , Bettin, KM , et al. Treatment of asymptomatic Clostridium difficile carriers (fecal excretors) with vancomycin or metronidazole. A randomized, placebo-controlled trial. Ann Intern Med 1992;117:297–302. 1322075 10.7326/0003-4819-117-4-297

[6] Turner, NA , Krishnan, J , Nelson, A , et al. Assessing the impact of 2-step Clostridioides difficile testing at the healthcare facility level. Clin Infect Dis 77:2023;1043–1049. 37279965 10.1093/cid/ciad334 PMC10552580

[7] Cho, DH , Si-Ho, K , Jeon, CH , et al. Clinical outcomes and treatment necessity in patients with toxin-negative Clostridioides difficile stool samples. Ann Clin Microbiol Antimicrob 2024;23:35. 38664689 10.1186/s12941-024-00696-1 PMC11046793

[8] Hogan, CA , Hitchcock, MM , Frost, S , et al. Clinical outcomes of treated and untreated *C. difficile* PCR-positive/toxin-negative adult hospitalized patients: a quasi-experimental noninferiority study. J Clin Microbiol 2022;60:6. 10.1128/jcm.02187-21 PMC9199396 35611653

